# Medication Adherence and Associated Factors for Children With Tic Disorders in Western China: A Cross-Sectional Survey

**DOI:** 10.3389/fneur.2019.01156

**Published:** 2019-11-05

**Authors:** Chunsong Yang, Wanxiang Qin, Dan Yu, Jiayuan Li, Lingli Zhang

**Affiliations:** ^1^Department of Epidemiology, West China School of Public Health, West China Fourth Hospital, Sichuan University, Chengdu, China; ^2^Department of Pharmacy, Evidence-Based Pharmacy Center, West China Second Hospital, Sichuan University, Chengdu, China; ^3^Key Laboratory of Birth Defects and Related Diseases of Women and Children (Sichuan University), Ministry of Education, Chengdu, China; ^4^Department of Pain Care, Southwest Hospital, Army Medical University, Chongqing, China; ^5^Department of Pediatric Neurology, West China Second Hospital, Sichuan University, Chengdu, China

**Keywords:** medication adherence, tic disorders, quality of life, children, western China

## Abstract

**Background:** Adherence to treatment remains important for successful tic disorder (TDs) management, but no studies had previously been carried out to assess adherence or the factors that affect it in children with TDs. This study therefore aimed to explore adherence to prescribed medication among children with tic disorders and to examine the associated factors.

**Methods:** This was a cross-sectional study in western China, where children with tic disorders were recruited consecutively in 2018. We used the eight-item Morisky Medication Adherence Scale (MMAS-8) to assess adherence. We included sociodemographic data, disease status, medication status, and family conditions as independent variables and used an ordinal logistic regression model to examine the factors affecting medication adherence.

**Results:** A total of 204 patients were included, with a response rate of 96.2%. The majority of participants (77.5%) were male, aged 7.69 ± 2.58 years. In total, 37.7% were newly diagnosed, 46.1% were review patients, and 16.2% were recurrent patients. Only 40.7% of patients showed high adherence (MMAS-8 score, 8). Decreasing quality of life (adjusted odds ratio 0.974) and living in non-rural areas (adjusted odds ratio 2.361) were significant independent determinants of non-adherence.

**Conclusion:** The results suggest that primary healthcare providers in pediatric clinics should focus on medication adherence counseling for children with tic disorders who have a lower quality of life and live in non-rural areas.

## Introduction

Tic disorders (TDs) are neurodevelopmental diseases characterized by fast, sudden, non-rhythmic, repetitive, and stereotyped phonic production and/or movements (1). Over two-thirds of individuals with TDs experience improvement in late adolescence, but some continue to have severe tics and need intensive treatment in adulthood (2). TDs are frequently comorbid with other conditions such as attention-deficit/hyperactivity disorder, obsessive–compulsive disorder, anxiety disorder, depression, and “rage attacks” (sudden, explosive episodes of rage) (3–5). A previous epidemiological study found that the prevalence was 2.99% for transient tic disorder, 1.615% for chronic tic disorder, and 0.77% for Tourette syndrome (6).

Caregivers of patients with TDs have been reported as having a tendency to develop psychological diseases (7). Both children and caregivers emphasize the importance of reducing tics, so appropriate treatment is a clinical priority.

Treatment approaches to TDs vary considerably with the severity and complexity of the clinical picture. Motor/vocal tics and comorbid symptoms are often managed by pharmacotherapy. The most common pharmacological treatments include antipsychotics, alpha-2 adrenoreceptor agonists, and antiepileptic drugs. Successful therapy for TDs can eliminate or reduce symptoms, so adherence to medication is important for treatment success. Adherence to medication is defined as the extent to which a person's behavior in taking medication corresponds with the agreed recommendation from a healthcare provider (8).

Adherence to treatment remains important for successful TD management. However, no studies had previously assessed adherence or the factors that affect it in children with TDs. This study, therefore, aimed to (1) assess adherence rates for children with TDs from western China and (2) identify factors (e.g., basic characteristics, disease status, medication status, and family situation) associated with non-adherence.

## Methods

### Study Design

This was a cross-sectional study using a questionnaire to assess adherence to prescribed medication for TDs in children. Based on the published literature and expert opinions, a questionnaire was designed by investigators to collect data in person. A pre-tested structured questionnaire was then administered by a trained pharmacist or doctor. Data were collected by investigators during face-to-face interviews lasting 15–20 min.

### Study Population

Eligible patients were consecutively selected by the physicians, who informed them of the study objectives and recruited all patients from the Pediatric Neurology clinic who agreed to participate between January and May 2019. The study was carried out in West China Second Hospital of Sichuan University (Sichuan Province, China). The hospital is the largest and most authoritative center for the diagnosis and treatment of childhood diseases in western China. It currently holds 1,580 beds and covers two districts. The hospital performs a large amount of clinical work, such as treatment, referral, and consultation for children with critical illnesses.

The inclusion criteria were: (1) a clinically confirmed diagnosis of TD, (2) the patient's written informed consent, and (3) age < 18 years. The exclusion criteria were: (1) delays or problems with mental development (Wechsler intelligence quotient [IQ] scores < 70 points), (2) presence of other chronic diseases (e.g., congenital heart disease, diabetes) that could affect adherence to therapeutic guidelines, and (3) lack of consent.

### Data Collection

A specifically-designed questionnaire was used to collect data. Children over 8 years old completed the questionnaire independently, and those under 8 years old were assisted by their guardians. The questionnaire covered four aspects, details of which are shown in Table 1.

**Table 1:** The five aspects of the designed questionnaire.

**Aspects**	**Variable**
Basic characteristics:	Name, gender, age
Disease status:	Time of disease, type of visit to the hospital, tic symptom, type of TD, family history, comorbidity, quality of life, regular review
Medication status	Type of medication, quantity of medication, beliefs about the necessity of taking medication, any concerns about taking medication
Family situation:	Parents' marital status, caregiver's relationship with child, caregiver's age, education level, place of residence, total household income, and medical expenses payment

### Instruments

Two validated instruments were used: the Beliefs about Medicines Questionnaire (BMQ) and the eight-item Morisky Medication Adherence Scale (MMAS-8).

#### Beliefs About Medicines Questionnaire (BMQ)

The BMQ was used to assess patients' beliefs about the medication prescribed for a particular illness. It is divided into two major subscales: “Necessity Beliefs” and “Concerns.” The Necessity Beliefs subscale assesses the patients' views on the necessity of taking the medication to maintain or improve their health. The Concerns subscale focuses on beliefs about the adverse effects of taking medication (9). Total scores for both subscales range from 5 to 25. Higher scores indicate stronger beliefs.

#### Medication Adherence

The Morisky Medication Adherence Scale (MMAS-8) was used to assess patients' medication adherence. This scale is widely used and has good reliability and validity (10, 11). MMAS-8 consists of eight questions with a scoring scheme of “Yes” = 0 and “No” = 1, and the items are summed to give a range of scores from 0 to 8. Higher scores indicate better adherence. This self-report measure is reported to have acceptable internal consistency and has demonstrated construct validity (12). Each item measures a specific medication-taking behavior, and the aggregate score indicates patients' inclination to adhere to their prescribed medication. Scores of 8 were considered to show high adherence, scores of 6–7 medium, and scores of 0–5 low (12). We attended a training and certification session for the Morisky Widget in August 2019 in Beijing, China, and obtained licenses for the use of MMAS-8 from MMAS Research LLC, USA.

### Data Analysis

The differences between adherent and non-adherent groups were investigated using analysis of variance for continuous variables. Quantitative variables are shown as mean and standard deviation, and qualitative variables as frequency and percentage. Categorical variables were analyzed using the chi-square or Fisher's exact test as appropriate. Quantitative variables were analyzed using variance analysis.

Following the univariate analysis, factors with *p* ≤ 0.05 were included in the multivariate logistic regression model. Multivariate analysis was used to examine the relative contribution of potential predictors of adherence categorization using ordered logistic regression. A *p* ≤ 0.05 was assumed to be statistically significant. Data analysis used SPSS version 22 (SPSS Inc., Chicago, IL, USA).

### Ethical Issues

The study was approved by the Office of Research Ethics Committees of West China Second Hospital. Written informed consent was obtained from all caregivers, and consent was obtained from children aged >8 years.

## Results

### Basic Characteristics

In total, 212 patients were clinically examined during the study period, of whom six refused to participate in the study and two showed mental development issues, giving a total of 204 patients (96.2%) who fulfilled the inclusion criteria.

Of these, 77.5% (158/204) were male, aged 7.69 ± 2.58 years, 37.7% (77/204) were newly diagnosed, 46.1% (94/204) were review patients, and 16.2% (33/204) were recurrent patients. Over half, 55.9% (114/204), only had motor tic symptoms, 5.9% (12/204) showed vocal tic symptoms, and 38.2% (78/204) showed both. In total, 45.1% (92/204) children had transient tic disorder, 31.9% (65/204) chronic tic disorder, and 23.0% (47/204) Tourette syndrome, while 9.8% (20/204) had a family history of TDs, and 18.1% (37/204) had comorbidities. The characteristics of the participants are shown in Table 2.

**Table 2:** Characteristics of the study population.

		**Low adherence**	**Medium adherence**	**High adherence**	**χ^2^ /F**	***P***
**Gender**						
	Male	54	38	66	0.790	0.674
	Female	19	10	17		
Age		7.57 ± 2.30	8.48 ± 2.93	7.35 ± 2.54	3.066	0.049
Time of disease		1.15 ± 1.11	1.75 ± 1.47	1.40 ± 1.38	2.724	0.068
**Type of visiting**						
	New diagnosis	29	15	33	4.207	0.379
	Review	30	28	36		
	Relapse	14	5	14		
**Tic symptom**						
	Motor tic	41	26	47	1.508	0.825
	Vocal tic	5	4	3		
	Both	27	18	33		
**Type of TDs**						
	TTD	36	17	39	3.337	0.503
	CTD	23	16	26		
	TS	14	15	18		
**Family history**						
	No	67	46	71	3.965	0.138
	Yes	6	2	12		
**Comorbidity**						
	No	62	37	68	1.202	0.548
	Yes	11	11	15		
Quality of life		80.69 ± 11.99	79.98 ± 12.03	85.31 ± 10.39	4.615	0.011
**Regular review**						
	No	5	4	3	1.469	0.480
	Yes	68	44	80		
**Type of medication**						
	Tiapride	30	27	35	13.915	0.177
	Clonidine	7	1	6		
	Vitamins	18	10	21		
	Tiapride+Clonidine +Vitamins	16	8	18		
	Tiapride+Clonidine +Haloperidol	2	0	3		
	Tiapride+Clonidine +Topiramate	0	2	0		
**Quantity of medication**						
	1	55	38	62	0.358	0.836
	≥2	18	10	21		
Necessity beliefs		3.03 ± 0.35	3.10 ± 0.36	3.02 ± 0.49	0.683	0.506
Concerns beliefs		3.36 ± 0.52	3.47 ± 0.46	3.26 ± 0.56	2.484	0.086
**Parents marital status**						
	Divorced	6	4	3	1.911	0.385
	Non-divorced	67	44	80		
**Caregiver**						
	Parents	70(37.6%)	39(21.0%)	77(41.4%)	7.382	0.026
	Non-parents	3(16.7%)	9(50.0%)	6(33.3%)		
**Caregivers' age**						
	<50 years	71(37.8%)	40(21.3%)	77(40.9%)	7.5	0.024
	≥50 years	2(12.5%)	8(50.0%)	6(37.5%)		
**Education level**						
	Below high school	19(29.7%)	24(37.5%)	21(32.8%)	10.125	0.006
	High school or above	54(38.6%)	24(17.1%)	62(44.3%)		
**Place of residence**						
	Rural	5(15.2%)	11(33.3%)	17(51.5%)	7.426	0.024
	Non-rural	68(39.8%)	37(21.6%)	66(38.6%)		
**Total household income/year**						
	≤ 12,000 yuan	26	17	30	0.008	0.996
	>12,000 yuan	47	31	53		
**Medical expenses payment**						
	Difficult to pay	0	2	2	3.894	0.143
	Able to pay	73	46	81		

### Adherence Rates

Of the 204 patients, 40.7% (83/204) had high medication adherence (scores of 8), 23.5% (48/204) had medium adherence (6.82 ± 0.11), and 35.8% (73/204) had low adherence (2.76 ± 2.52).

### Factors Associated With Adherence (Tables 2, 3)

#### Patient-Related Factors

The univariate analysis identified two patient-related factors that were significantly associated with adherence. These were age of patient (*F* = 3.066, *P* = 0.049) and quality of life (*F* = 4.615, *P* = 0.011).

**Table 3:** Ordered Logistic regression analysis of influencing factors of medication adherence in patients with TDs.

	**Variable**	**B**	**SE**	**Wald**	**P**	**OR**	**95% CI**
Age		−0.031	0.055	0.319	0.572	0.969	0.871–1.079
Quality of life		0.027	0.012	4.913	0.027	1.027	1.003–1.051
Caregiver	Parents	0.453	0.820	0.305	0.581	1.573	0.315–7.854
	Non-parents						
Caregivers' age	<50 years	−0.994	0.862	1.329	0.249	0.370	0.068−2.006
	≥50 years						
Education level	Below high school	−0.227	0.339	0.450	0.502	0.797	0.410–1.548
	Below high school						
Place of residence	Rural	0.859	0.404	4.519	0.034	2.361	1.069–5.212
	Non-rural						

#### Medication-Related Factors

The univariate analysis found no medication-related factors associated with adherence.

#### Family-Related Factors

The univariate analysis found four caregiver-related factors associated with adherence. These were the relationship between caregiver and child (χ^2^ = 7.382, *P* = 0.026), caregiver's age (χ^2^ = 10.125, *P* = 0.006), education level (χ^2^ = 10.125, *P* = 0.006), and place of residence (χ^2^ = 7.426, *P* = 0.024).

The six significant factors from the univariate analysis were included in the multivariate logistic regression model. The results showed that quality of life and place of residence were associated with adherence. Increasing quality of life (odds ratio [OR] = 1.027, 95% confidence interval [CI] 1.003–1.051) was associated with high adherence, and living in non-rural areas (OR = 2.361, 95% CI 1.069–5.212) was more likely to be associated with low adherence.

## Discussion

### Statement of Main Findings

This study is the first of which we are aware to use a self-reported questionnaire to assess adherence to medication in children with TDs. The results showed that only 40.7% of patients with TDs were adhering to medication guidelines. We also found that quality of life and place of residence were associated with adherence. This may be because patients with a higher quality of life have a more positive attitude to TDs and are more inclined to follow the doctor's advice. Children living in rural areas may be more compliant because it is inconvenient to seek medical treatment, so it is valued more. Their overall economic situation is also relatively low, so again, they are more inclined to value medication and use it correctly.

There have been no other studies assessing adherence to medication in children with TDs, so we could not compare our findings. However, a systematic review that evaluated medication adherence among children with epilepsy found that over half of patients worldwide adhered to medication guidelines when using an objective method to assess adherence, which is similar to our finding, but the pooled adherence rate for children assessed by subjective methods was 73%, which is higher than our finding (13). Compared with the adherence to treatment for asthma management, our finding is also similar to the population-based cohort study conducted in a Dutch primary care database containing medical records of 176,516 children aged 5–18 years. The authors used the medication possession ratio (MPR) to evaluate adherence and reported good adherence (MPR > 0.8) in almost 46% of the leukotriene receptor antagonist users and in 34% of those using a fixed combination of inhaled corticosteroids and long-acting ß2-agonists (14). Another study reported the overall adherence rate to 6- Mercaptopurine in children and adolescents with Acute Lymphoblastic Leukemia as 80.8% (15), which was higher than our finding. This may be because the disease was very serious, so parents attached great importance to the disease, and so the overall adherence rate was high. Our results showed that low medication adherence in children with TDs from western China is a prevalent problem.

Adherence to medication is important for effective disease management, so clinicians should try to improve it using a variety of interventions such as the following. (1) Clinicians should focus on quality of life and place of residence for patients when prescribing medicines for TDs, especially for patients with low adherence, because if they are not achieving good tic symptom control, this will aggravate the severity of the disease and affect the prognosis. It is therefore necessary to provide health education to guardians and caregivers to help them understand the condition, etiology, treatment, and precautions to take. (2) Clinicians should establish a follow-up system to detect patient medication compliance. (3) New forms of interventions and interactions, including text messaging, application-based interventions, and WeChat use, should be encouraged.

This study had some limitations. In particular, the limited sample size means that larger, multisite studies of medication adherence in children with TDs are needed to confirm these conclusions. Future prospective research should be designed with longer study periods and larger samples in naturalistic settings. It would also be helpful to examine the effectiveness of pharmacist-led intervention in improving medication adherence and to explore various forms of patient education. Future research may also use information technology, wearable devices, and other technologies to examine medication compliance.

The limitations of this study also need to be considered when interpreting its findings. First, there is no standardized tool for adherence measurement in patients with TDs. The Morisky scale is widely used and has good reliability and validity, but it is a self-reported measure that may therefore overestimate the prevalence of medication adherence. It is therefore necessary to use other objective methods to confirm these results. Second, selection bias might have occurred, because those who agreed to participate are likely to be more actively engaged with their condition and will therefore typically care more about their health. This may lead to a higher level of adherence. Third, the cross-sectional study design carries limited possibilities for exploring causal relationships and does not provide clinical follow-up. Further studies are needed to overcome these shortcomings.

## Conclusion

Low medication adherence is a prevalent problem in children with TDs. Primary healthcare providers in pediatric clinics should focus on medication adherence counseling for children with TDs with a lower quality of life and who live in non-rural areas.

## Data Availability Statement

The raw data supporting the conclusions of this manuscript will be made available by the authors, without undue reservation, to any qualified researcher.

## Ethics Statement

The studies involving human participants were reviewed and approved by the Office of Research Ethics Committees of West China Second Hospital. Written informed consent to participate in this study was provided by the participants' legal guardian/next of kin.

## Author Contributions

CY designed the review, collected data, carried out analysis and interpretation of the data, and wrote the review. WQ and DY designed the review, collected data, checked the data, and wrote the review. JL and LZ designed the review and commented on drafts of a previous version.

### Conflict of Interest

The authors declare that the research was conducted in the absence of any commercial or financial relationships that could be construed as a potential conflict of interest.
